# Mental and physical health morbidity among people in prisons: an umbrella review

**DOI:** 10.1016/S2468-2667(24)00023-9

**Published:** 2024-03-27

**Authors:** Louis Favril, Josiah D Rich, Jake Hard, Seena Fazel

**Affiliations:** a Institute for International Research on Criminal Policy, Faculty of Law and Criminology, Ghent University, Ghent, Belgium; b Warren Alpert Medical School, Brown University, Providence, RI, USA; c HM Prison Cardiff, Cardiff, UK; d Department of Psychiatry, University of Oxford, Oxford, UK; e Oxford Health NHS Foundation Trust, Oxford, UK

## Abstract

**Background:**

People who experience incarceration are characterised by poor health profiles. Clarification of the disease burden in the prison population can inform service and policy development. We aimed to synthesise and assess the evidence regarding the epidemiology of mental and physical health conditions among people in prisons worldwide.

**Methods:**

In this umbrella review, five bibliographic databases (Web of Science, PubMed, PsycINFO, Embase, and Global Health) were systematically searched from inception to identify meta-analyses published up to Oct 31, 2023, which examined the prevalence or incidence of mental and physical health conditions in general prison populations. We excluded meta-analyses that examined health conditions in selected or clinical prison populations. Prevalence data were extracted from published reports and study authors were contacted for additional information. Estimates were synthesised and stratified by sex, age, and country income level. The robustness of the findings was assessed in terms of heterogeneity, excess significance bias, small-study effects, and review quality. The study protocol was pre-registered with PROSPERO, CRD42023404827.

**Findings:**

Our search of the literature yielded 1909 records eligible for screening. 1736 articles were excluded and 173 full-text reports were examined for eligibility. 144 articles were then excluded due to not meeting inclusion criteria, which resulted in 29 meta-analyses eligible for inclusion. 12 of these were further excluded because they examined the same health condition. We included data from 17 meta-analyses published between 2002 and 2023. In adult men and women combined, the 6-month prevalence was 11·4% (95% CI 9·9–12·8) for major depression, 9·8% (6·8–13·2) for post-traumatic stress disorder, and 3·7% (3·2–4·1) for psychotic illness. On arrival to prison, 23·8% (95% CI 21·0–26·7) of people met diagnostic criteria for alcohol use disorder and 38·9% (31·5–46·2) for drug use disorder. Half of those with major depression or psychotic illness had a comorbid substance use disorder. Infectious diseases were also common; 17·7% (95% CI 15·0–20·7) of people were antibody-positive for hepatitis C virus, with lower estimates (ranging between 2·6% and 5·2%) found for hepatitis B virus, HIV, and tuberculosis. Meta-regression analyses indicated significant differences in prevalence by sex and country income level, albeit not consistent across health conditions. The burden of non-communicable chronic diseases was only examined in adults aged 50 years and older. Overall, the quality of the evidence was limited by high heterogeneity and small-study effects.

**Interpretation:**

People in prisons have a specific pattern of morbidity that represents an opportunity for public health to address. In particular, integrating prison health within the national public health system, adequately resourcing primary care and mental health services, and improving linkage with post-release health services could affect public health and safety. Population-based longitudinal studies are needed to clarify the extent to which incarceration affects health.

**Funding:**

Research Foundation–Flanders, Wellcome Trust, National Institutes of Health.

## Introduction

Worldwide, more than 11 million individuals are incarcerated on any given day.^1^ The life trajectories of people who experience incarceration are typically characterised by poor educational attainment, unemployment, unstable housing, poverty, and trauma—social determinants that negatively affect health.^2^ Compared with the general population, people living in prisons, jails, and juvenile detention facilities (collectively referred to as prisons hereafter) disproportionately experience mental health problems, substance misuse, infectious diseases, and chronic conditions.^3^, ^4^, ^5^, ^6^, ^7^ Poor mental health in prison populations is linked to a wide range of adverse outcomes, including reoffending, victimisation, and self-harm.^7^, ^8^, ^9^ Mortality rates are higher for people in prisons than for their community peers,^10^ especially from external causes such as suicide,^11^ and health outcomes after release from custody are notably poor.^12^ Because many people in prisons do not access primary care in the community,^13^ incarceration often represents the first opportunity to assess, diagnose, and treat health needs in this underserved group. Because of the large number of individuals who transition through prisons annually, which is estimated to be about 30 million people worldwide,^14^ improving the health of this vulnerable population is central to reducing health inequalities and to improving public health.^5^

**Research in context**

**Evidence before this study**

We searched Web of Science, PubMed, PsycINFO, Embase, andGlobal Health for umbrella reviews, published from database inception to Oct 31, 2023, without language limitations, using the search terms: (prison* OR jail* OR inmate* OR incarcerat* OR imprison* OR remand* OR sentence* OR detain* OR detention OR offend* OR custod*) AND (umbrella OR meta*). Although many health conditions have been the focus of individual systematic reviews and meta-analyses, we did not identify any umbrella reviews examining the overall health status of people in prisons.

**Added value of this study**

In this umbrella review of meta-analyses, we have presented a comprehensive overview of the mental and physical health morbidity among people in prisons and appraised the quality of the evidence base. We identified high prevalences of mental illness, substance misuse, and infectious diseases, which were at least double the rate in the general population. Drug use disorders, post-traumatic stress disorder, and sexually transmitted infections were more common in women than in men. However, findings should be interpreted in light of high heterogeneity and small-study effects.

**Implications of all the available evidence**

The available evidence underscores that people in prisons experience a higher burden of mental health conditions and physical illnesses than observed in the general population. Evidence gaps limit our knowledge of the full range and complexity of health needs in prisoners, particularly relating to non-communicable chronic diseases. Addressing the health needs of incarcerated people has the potential to improve public health and safety.

Effective service and policy development requires a comprehensive understanding of the disease burden in prisons, but recent evidence regarding the health of people who experience incarceration has, to our knowledge, not been fully synthesised. A large number of meta-analyses, of varying quality and samples, have provided prevalence estimates for specific health conditions.^5^ However, there remains a need to bring together the totality of the evidence to allow for a comparison of different conditions across mental and physical health domains; examine potential differences by sex, age, and country income level; evaluate the quality of the underlying evidence; and identify gaps in the literature. Therefore, in this umbrella review, we aimed to summarise and assess the meta-analytic evidence on the epidemiology of mental and physical health conditions among people in prisons worldwide. Findings could support clinical services in prioritising interventions, policy makers in allocating resources, and researchers in addressing evidence gaps.

## Methods

### Search strategy and selection criteria

We conducted an umbrella review^15^ to systematically collect and review published meta-analyses examining the prevalence and incidence of mental and physical health conditions in prison populations.

We did a title and abstract search in five electronic databases (Web of Science, PubMed, PsycINFO, Embase, and Global Health) for meta-analyses published from database inception to May 12, 2023, with no language restrictions. Our initial search was updated to include meta-analyses published until Oct 31, 2023. The same search string was used for each database search: (prison* OR jail* OR inmate* OR incarcerat* OR imprison* OR remand* OR sentence* OR detain* OR detention OR offend* OR custod*) AND (prevalence OR inciden* OR epidemiol*) AND (meta* OR “systematic review”). We used forward citation chaining to supplement our search, and reference lists of relevant reviews^4^, ^5^, ^6^, ^7^ were manually searched. We conducted additional searchees for grey literature in Google Scholar.

Eligible studies for inclusion were systematic reviews with meta-analysis that provided a pooled prevalence or incidence estimate of any mental or physical health condition among unselected correctional populations residing in prisons, jails, and juvenile detention facilities (collectively referred to as prisons). Systematic reviews without meta-analysis were not considered as we intended to provide quantitative comparisons and rate quality. Eligibility was assessed by LF and discussed with SF until a consensus was achieved.

Because the focus was on the general prison population, we excluded meta-analyses that examined health conditions in selected or clinical prison populations (eg, sex offenders, people who inject drugs, and those referred for treatment or with a specific diagnosis)^16^, ^17^, ^18^ or pooled prevalence across prisons and other closed settings (eg, forensic psychiatric units, immigration detention centres, and compulsory drug detention facilities).^19^, ^20^, ^21^, ^22^ We re-analysed the data for attention-deficit hyperactivity disorder^22^ (ADHD) by including solely unselected samples of adults in prisons.^23^ Justice-involved individuals not residing in prisons (such as formerly incarcerated people and those serving community sentences)^24^ were beyond the scope of this review. Meta-analyses that were restricted to a single country were also excluded because these would limit the generalisability of the findings.

We only considered health conditions that were assessed by clinical investigation (eg, biological markers for certain infectious diseases) or established with validated diagnostic instruments using a clinical or research interview (eg, semi-structured diagnostic interviews for mental disorders). For example, we did not include meta-analyses of mental health conditions based on screening tools because rates of false positives are high and these measures are not equivalent to clinical diagnoses. Substance use irrespective of meeting diagnostic criteria was also excluded.^25^

If more than one eligible meta-analysis was identified on the same health condition, we only retained one to avoid duplication of underlying samples.^15^ In this case, we selected the meta-analysis with the highest methodological quality, provided that individual-level study estimates were available. Review quality was assessed by LF and SF. There were no marked differences in prevalence estimates between these overlapping meta-analyses. For post-traumatic stress disorder (PTSD), we included the 6-month point prevalence estimate.^26^

### Data extraction and analysis

Data were extracted by LF using a standardised form and were cross-checked by SF for mental health conditions and by JH for physical health. Any disagreements were resolved by discussion between these three authors. For each eligible meta-analysis, we recorded the characteristics (country and date of publication) of primary studies, the number of samples included in the meta-analysis (k), pooled sample size (n), sample distribution by age and sex, individual prevalence estimates of primary studies, and the pooled prevalence estimate with 95% CI and corresponding heterogeneity statistic (*I*^2^) from random-effects meta-analysis. An *I*^2^ value (which describes the percentage of variability in prevalence estimates that is due to between-study heterogeneity) of less than 50% was taken to indicate low heterogeneity. Authors were contacted if study characteristics were unclear or when study-level estimates were not reported in the paper. When available, we extracted prevalence estimates for men and women separately. In case only sex-specific estimates were reported,^26^, ^27^, ^28^ we calculated an overall estimate (men and women combined) using random-effects models. Sex data were based on information reported in the meta-analyses.

The main analysis focused on individual health conditions among men and women of all ages. In a secondary analysis, we additionally synthesised data on specific age groups, low-income and middle-income countries (LMICs), and comorbidity.

Consistent with previous umbrella reviews,^29^ we synthesised findings from the included meta-analyses using a descriptive approach. Therefore, we report prevalence estimates (with corresponding 95% CIs and *I*^2^ statistic) and results from meta-regression analyses in the manner in which they were reported in the underlying meta-analyses. However, for three health conditions, we did re-calculate estimates based on prevalence data reported in the paper because the original reviews^27^, ^30^ did not adopt random-effects models, which would limit comparisons with other estimates.

For health conditions included in the main analysis, two additional analyses were conducted to assess the robustness and consistency of the evidence. First, we assessed if there was evidence for small-study effects (ie, whether smaller studies yield a higher prevalence than do larger studies) using the regression asymmetry test proposed by Egger.^31^ A p value less than 0·10 provides evidence for small-study effects. Second, the ratio between the pooled overall prevalence estimate of a meta-analysis and that of its largest included primary study (assumed to be the most accurate) was calculated as a measure of statistical excess bias. A ratio greater than one is an indication of excess significance.

We additionally calculated 95% prediction intervals for each health condition to provide an estimate of the range in which future observations will fall.^32^

The methodological quality of included meta-analyses was rated using the Risk of Bias in Systematic Reviews (ROBIS) instrument.^33^ ROBIS is a tool specifically designed to assess the risk of bias in systematic reviews, which is rated on four domains: study eligibility criteria (five items), identification and selection of studies (five items), data collection and study appraisal (five items), and synthesis and findings (six items). Based on these domain ratings, we computed an overall risk of bias score, classifying each meta-analysis as having low, moderate, or high risk of bias.^29^

We used an overall quality assessment developed for umbrella reviews.^29^ Each identified health condition was assigned a score of 0 (low quality) or 1 (high quality) on four criteria: between-study heterogeneity, small-study effects, excess significance bias, and review quality. The four quality scores were then summed to determine an aggregate quality rating within the range of 0–4, with 0 indicating the lowest overall quality and 4 indicating the highest quality.

All analyses were done with Stata (version 13). The study was registered on PROSPERO, CRD42023404827. There were no deviations from the protocol and we followed PRISMA guidelines (appendix pp 2–4).^34^

### Role of the funding source

The funders of the study had no role in study design, data collection, data analysis, data interpretation, or writing of the report.

## Results

Our systematic search of the literature yielded 1909 records eligible for screening. Following the exclusion of 1736 articles based on title and abstract, 173 full-text reports were examined for eligibility. 144 articles were subsequently excluded on the basis of design, population, and outcome assessment, resulting in 29 meta-analyses that met our inclusion criteria. 12 of these were further excluded because they examined the same health condition, resulting in 17 meta-analyses being included in our review (appendix pp 5–7). No additional eligible studies were identified in the updated search.

12 meta-analyses reported prevalence data on 18 health conditions in incarcerated men and women across age groups, which were included in the main analysis (table 1).^23^, ^26^, ^27^, ^30^, ^35^, ^36^, ^37^, ^38^, ^39^, ^40^, ^41^, ^42^ We additionally identified three meta-analyses restricted to specific age groups (ie, adolescents^28^ and older adults^43^, ^44^), one on comorbidity of mental disorders,^45^ and one limited to LMICs only.^46^ Given their specific focus, findings from these five meta-analyses are discussed separately in a secondary analysis.

**Table 1:** Prevalence of mental and physical health conditions among people in prisons

	**k**	**n**	**Period**	**Prevalence (95% CI)**	I**^2^**	**Prediction interval**	**Small-study effects (p)**	**Excess significance**	**Risk of bias**	**Overall quality**
**Mental health condition**										
Psychotic illness^35^	99	30 635	6 months	3·7% (3·2–4·1)	88	0·1–7·6	<0·0001	0·6	Moderate	1
Major depression^35^	74	20 049	6 months	11·4% (9·9–12·8)	95	3·0–22·3	0·010	0·8	Moderate	1
Post-traumatic stress disorder^26^	50	19 011	6 months	9·8% (6·8–13·2)	98	2·5–17·8	<0·0001	32·7	Low	1
Alcohol use disorder^36^	24	17 656	12 months	23·8% (21·0–26·7)	94	9·7–38·0	0·060	0·8	Low	2
Drug use disorder^36^	23	10 612	12 months	38·9% (31·5–46·2)	99	1·5–76·3	0·033	0·7	Low	2
Attention-deficit hyperactivity disorder^23^	11	3919	Current	8·3% (3·8–12·8)	95	0–23·8	0·011	3·6	Moderate	0
Antisocial personality disorder^27^	28	13 844	Lifetime	40·4% (31·8–49·0)	99	0·8–80·0	0·215	0·7	High	2
Borderline personality disorder^27^*	5	1208	Lifetime	22·7% (17·8–27·7)	64	6·8–38·7	0·183	0·8	High	2
**Physical illness**										
Hepatitis C virus^37^	93	145 823	Lifetime†	17·7% (15·0–20·7)	99	3·2–43·6	<0·0001	5·5	High	0
Hepatitis B virus^38^	31	61 867	Lifetime†	5·2% (2·2–9·3)	100	0–17·3	0·030	26·0	High	0
HIV^39^	72	2 275 930	Current	3·4% (3·2–3·6)	99	0–12·5	<0·0001	2·3	High	0
Tuberculosis^40^	59	1 012 448	Current	2·6% (2·1–3·3)	100	0·7–7·4	<0·0001	0·4	Low	2
Chlamydia^41^	39	381 374	Current	8·9% (8·2–9·7)	99	1·9–15·8	<0·0001	1·3	Low	1
Gonorrhoea^41^	30	121 448	Current	3·3% (2·9–3·8)	96	0·5–6·4	<0·0001	2·4	Low	1
Syphilis^41^	30	439 838	Current	2·9% (2·6–3·2)	97	1·1–6·3	<0·0001	5·2	Low	1
Human papillomavirus^42^*	9	1322	Current	29·8% (20·0–39·5)	94	0–65·8	0·027	1·1	Moderate	0
Cervical intraepithelial neoplasia^42^*	22	46 026	Current	8·4% (6·7–10·1)	96	0·8–16·0	0·075	1·7	Moderate	0
Epilepsy^30^	7	3111	Lifetime	0·6% (0·3–0·8)	0	0·2–0·9	0·074	1·6	High	1

Pooled prevalence estimates (with CIs and *I*^2^) are from random-effects models, as reported in the original meta-analyses. Data refer to men and women combined unless otherwise stated. Risk of bias was assessed by ROBIS and rated as high, moderate, or low. Overall quality scores range from 0 to 4, with higher scores indicating higher quality. k=number of samples included in the meta-analysis. n=pooled sample size.

* Women only.

† Past or current infection.

Meta-analyses included in the main analysis were published between 2002 and 2023. The proportion of study samples from LMICs was 26% (range 0–78) across the 12 meta-analyses. Of the meta-anlayses on physical health, 42% (135 of 320) of the underlying samples were from LMICs and of those regarding mental health, 9% (27 of 314) were from LMICs. A total of 39 LMICs were represented (appendix p 8). Two meta-analyses were restricted to data from high-income countries (HICs) only.^27^, ^36^ The number of samples analysed for each health condition ranged from five to 99, with a median of 30 (IQR 22–57). All mental health conditions were established by a diagnostic or clinical interview, and all physical health conditions (except for epilepsy) included biological or serological markers as part of their diagnosis.

Based on ROBIS criteria, four (33%) of the 12 meta-analyses were rated as low risk of bias, three (25%) as moderate risk, and five (42%) as high risk. Common limitations were the absence of a pre-registered study protocol (50%; six of 12), no risk-of-bias assessment of primary studies (58%; seven of 12), and insufficient examination of sources of heterogeneity (42%; five of 12). Other quality tests performed at the level of individual health conditions also found indications of poor quality. Heterogeneity was high (*I*^2^>50%) for all but one (94%) of the prevalence estimates. 16 (89%) of the 18 health conditions had small-study effects and 11 (61%) demonstrated evidence of excess significance (more clearly in the physical domain [90%; nine of ten] compared with the mental domain [25%; two of eight]). Overall quality ratings (based on four criteria) indicated mostly low quality. The mean quality score across all health conditions was 0·9 (out of 4) with higher mean scores for mental (1·4) than physical (0·6) health conditions. No health condition met full criteria. With composite scores less than 2, quality was low for 72% (13 of 18) of all conditions examined.

**Figure 1:**
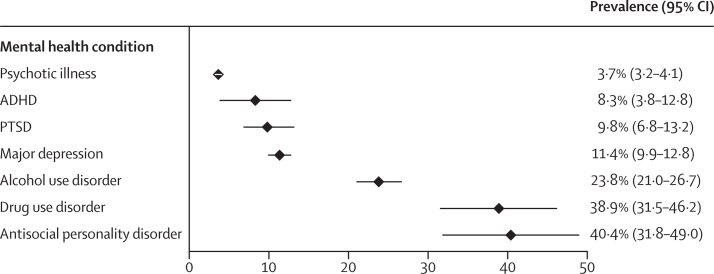
Prevalence of mental disorders among males and females in prisons Data are prevalence (%) and 95% CI. ADHD=attention-deficit hyperactivity disorder. PTSD=post-traumatic stress disorder.

We identified five meta-analyses reporting on eight mental health conditions (table 1; appendix pp 9–13). The overall prevalence of mental disorders ranged from 3·7% (95% CI 3·2–4·1) for psychotic illness to 40·4% (31·8–49·0) for antisocial personality disorder (figure 1). Major depression (11·4% [95% CI 9·9–12·8]) and PTSD (9·8% [6·8–13·2]) were estimated to affect one in every ten people in prisons. In admission samples, 23·8% (95% CI 21·0–26·7) of recently incarcerated adults met diagnostic criteria for alcohol use disorder and 38·9% (31·5–46·2) for drug use disorder (table 1). In women, the prevalence of borderline personality disorder was estimated at 22·7% (95% CI 17·8–27·7). For meta-analyses that stratified analyses by sex, the prevalence of most mental disorders was higher in women than in men (table 2). In meta-regression analyses, there was no significant difference in the prevalence of psychotic illness, major depression, and alcohol use disorder between men and women, whereas women had a higher prevalence of PTSD and drug use disorder than did men (table 2). Antisocial personality disorder was significantly more common in men (45·8% [95% CI 39·5–52·1]) than in women (27·2% [16·2–38·3]). In the only two meta-analyses that compared samples from both LMICs and HICs, meta-regression indicated a significantly higher prevalence of psychotic illness and major depression in LMICs compared with HICs, whereas PTSD was more common in HICs than in LMICs (appendix p 14).

**Table 2:** Prevalence of mental disorders among people in prisons by sex

	**Males**	**Females**
Psychotic illness^35^	3·6% (3·1–4·2)	3·9% (2·7–5·0)
Major depression^35^	10·2% (8·8–11·7)	14·1% (10·2–18·1)
Post-traumatic stress disorder^26^*	6·2% (3·9–9·0)	21·1% (16·9–25·6)
Alcohol use disorder^36^	26·2% (22·6–29·8)	20·0% (16·2–23·7)
Drug use disorder^36^*	29·7% (21·6–37·8)	50·7% (43·4–58·1)
Antisocial personality disorder^27^*	45·8% (39·5–52·1)	27·2% (16·2–38·3)

Pooled prevalence estimates (with 95% CI) as reported in the original meta-analyses.

* Significant sex difference reported in meta-regression analyses of the original reviews.

Four additional meta-analyses that specifically focused on mental disorders in adolescents,^28^ people aged 50 years and older,^43^ LMICs,^46^ and comorbidity^45^ were included in a secondary analysis, for which the main findings are summarised in the appendix (pp 15–18).

**Figure 2:**
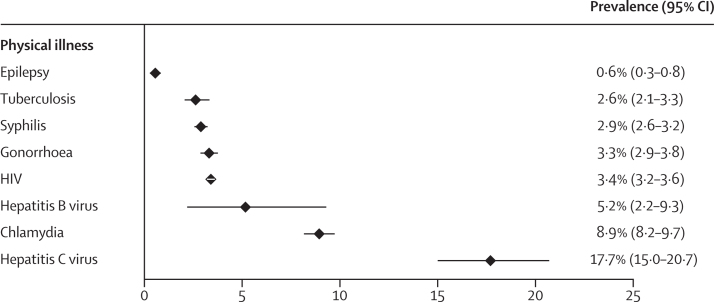
Prevalence of physical health conditions among males and females in prisons Data are prevalence (%) and 95% CI.

We identified seven meta-analyses reporting on ten physical health conditions, most of which were infectious diseases (table 1). Prevalences ranged from 0·6% (95% CI 0·3–0·8) for epilepsy to 17·7% (15·0–20·7) for hepatitis C virus (figure 2). Other blood-borne viruses were less common; 5·2% (95% CI 2·2–9·3) for hepatitis B virus and 3·4% (3·2–3·6) for HIV. A meta-analysis of 60 studies on bacterial sexually transmitted infections found a point prevalence of 8·9% (95% CI 8·2–9·7) for chlamydia, 3·3% (2·9–3·8) for gonorrhoea, and 2·9% (2·6–3·2) for syphilis, with meta-regression indicating significantly higher prevalences in women than in men for all three conditions.^41^ Prevalence estimates stratified by sex were not available for most other physical health conditions (appendix p 19). Drawing on data from 53 533 women in prisons,^42^ the prevalence of human papillomavirus infection was 29·8% (95% CI 20·0–39·5) and the prevalence of cervical intraepithelial neoplasia was 8·4% (6·7–10·1; table 1). Although most meta-analyses on physical health conditions did not examine potential differences by country income level, the prevalence of active tuberculosis was largely similar in LMICs (3·1% [95% CI 1·8–5·3]) and HICs (2·3% [1·7–3·0]), with an overall prevalence of 2·6% (2·1–3·3).

In a secondary analysis, we additionally identified one meta-analysis^44^ on non-communicable chronic diseases (eg, cancer, hypertension, diabetes, and asthma) in adults aged 50 years and older (appendix p 20). Relative to the general population, the prevalence of most mental and physical health conditions was reported to be substantially higher among people in prisons (appendix p 21).

## Discussion

In this umbrella review of 17 meta-analyses published over two decades, we have provided a comprehensive overview of the disease burden among people in prisons worldwide. Findings indicate that incarcerated individuals experience poor health across a wide range of mental and physical conditions. However, prevalence estimates should be interpreted in light of high heterogeneity, small-study effects, and risk of bias in the underlying meta-analyses. We report four main results.

First, the burden of treatable mental disorders among incarcerated individuals is substantial. One in every ten people was diagnosed with depression (11%) or PTSD (10%), and psychotic illness affected about 4% of the prison population. A quarter (24%) of people who enter prison were found to have an alcohol use disorder and 39% a drug use disorder. These mental health conditions rarely present in isolation; around half of people in prisons with depression (52%) or psychotic illness (49%) had a comorbid substance use disorder. Meta-regression analyses further pointed to important differences in prevalence by sex and country income level. Drug use disorder and PTSD were more common in women than in men, with no clear sex differences in depression, psychotic illness, and alcohol use disorder. By country income level, psychotic illness and depression were more common in LMICs, whereas PTSD was more prevalent in HICs. In relation to age, the prevalence of most mental disorders was found to be largely similar for adolescents and adults, except for ADHD which was more common in adolescents. Overall, when compared with the general population, most mental disorders were at least twice as prevalent among people in prisons.

Second, our findings indicate a high prevalence of infectious diseases. Around one in six (18%) people in prisons had a current or past hepatitis C virus infection, with relatively lower estimates found for hepatitis B virus (5%), HIV (3%), and tuberculosis (3%). Bacterial sexually transmitted infections such as chlamydia (9%) were also common, with higher rates in women than in men. Among people in prison aged 50 years and older, non-communicable diseases including hypertension (39%), diabetes (14%), and asthma (7%) were generally more prevalent compared with their younger peers in prisons. Meta-regression analyses examining potential differences in prevalence by sex and country income level were not available for most physical health conditions.

Third, pooled prevalence estimates should be interpreted in the context of small-study effects and high heterogeneity, which were present in many meta-analyses contributing to this umbrella review. Small-study effects might indicate publication bias, which is likely to lead to inflated prevalence estimates. The high level of heterogeneity (with *I*^2^>90% for most conditions) might be due to the primary studies being conducted in a large variety of prison settings and expected changes in prevalence over time (eg, due to policy reforms). Prison populations are likely to vary substantively between countries owing to differences in national policies regarding management of health conditions within the criminal justice system (eg, alternative sentencing and diversion strategies), which might further contribute to heterogeneity. For example, in some countries, community sentences can provide an alternative to custody,^47^ which allows for treatment of mental illnesses and substance misuse. Prevalence ranges should thus be considered as alternatives to pooled estimates. Furthermore, around two-thirds of meta-analyses included in our review had moderate or high risk of bias, with common limitations including insufficient consideration of heterogeneity and bias in primary studies. Future reviews should therefore consider possible sources of heterogeneity more carefully, as per methodological guidelines,^34^ and aim to meet other quality criteria.

Finally, this umbrella review highlights several key gaps in the meta-analytic evidence-base. First, none of the included meta-analyses examined incidence rates. Although not eligible for inclusion, one recent example exists for tuberculosis,^21^ for which the incidence per 100 000 person-years was 260 in jails and 450 in prisons (ten times higher than in the general population). Overall, accurate information on incidence rates, including re-infection rates of viral hepatitis, is needed to complement prevalence data and provide a more complete picture of the disease burden in prisons. Second, there was a notable lack of evidence on the prevalence of non-communicable diseases such as cancer,^48^ diabetes,^49^ and cardiovascular disease.^50^ The only identified meta-analysis in this area was restricted to older adults.^44^ Comparable data on young adults, who constitute the large majority of the prison population worldwide, would be informative in the context of the reported accelerated ageing of people in prisons.^51^ Third, meta-analyses on epilepsy and personality disorders were published more than 20 years ago and thus require updating, for example, by including prevalence data on borderline personality disorder in men.^52^ Fourth, the global burden of bipolar disorder, anxiety disorders, eating disorders, and autism spectrum disorder in prison populations has not yet been meta-analytically reviewed to date, whereas intellectual disability^53^ and traumatic brain injury^54^ require further investigation based on reliable and clinically informative diagnostic criteria. Fifth, we did not identify any meta-analyses examining the comorbidity between mental and physical health conditions.^55^ Together, these evidence gaps limit our knowledge of the full range and complexity of health needs among people in prisons.

Strengths of this umbrella review include synthesising a broad range of health conditions and using methodological tests to assess the quality of evidence. However, there are also several limitations. First, our findings only apply to health conditions in general prison populations and might not be generalisable to selected and high-risk groups, in which prevalence is likely to be higher.^17^ Second, because we only considered health conditions that have been subject to meta-analysis, other health conditions reported in narrative or systematic reviews (without quantitative synthesis) were not included in our overview.^48^, ^49^, ^50^ Third, when multiple eligible meta-analyses evaluated the same health condition, we retained the one with the highest quality, provided that individual-level study estimates were available. This latter criterion led to findings on infectious diseases being based on meta-analyses of low quality. Fourth, our global scope might have masked important differences between countries and regions in terms of prevalence, resources, and policies.

The nature of the relationship between incarceration and health remains a key question for public health and policy—is incarceration a marker of pre-existing health inequalities or a cause of poor health outcomes? On the one hand, there is epidemiological evidence supporting the selection hypothesis in that mental illness and substance misuse are important risk factors for criminal offending and incarceration,^56^, ^57^, ^58^ by which pre-existing health morbidity is imported into prison. Additionally, people in prisons often have histories of homelessness^59^ and childhood adversity,^60^ which make them vulnerable to experiencing poor health.^61^, ^62^, ^63^ Other research highlights selection processes that disproportionately funnel people from disadvantaged backgrounds into prisons,^64^ which contributes to health inequalities. On the other hand, incarceration confers its own unique risks that negatively impact on health, including exposure to a stressful evironment.^65^ Health-risk behaviours such as smoking,^66^ poor diet, and physical inactivity^67^ are common during incarceration and might impair health or exacerbate underlying conditions. Drug injection, unsafe sexual activity, and tattooing^68^ specifically contribute to in-prison transmission of viral infections.^69^ Furthermore, environmental conditions of confinement such as overcrowding, lack of sanitation and hygiene, and poor ventilation are conducive to the spread of infectious diseases, with COVID-19 being a topical example.^70^ Wider social and organisational issues related to the prison regime, including isolation and limited opportunities for purposeful activity,^71^ are known to negatively affect mental health.^72^ By contrast, incarceration might also confer a temporary health benefit to some individuals by decreasing exposure to behavioural and social risk factors (eg, substance misuse and victimisation) and providing access to health care.^73^ For people from marginalised backgrounds, living conditions in prisons (eg, shelter and regular meals) might be an improvement over their standards before entering prison,^73^ which can have a health-promoting effect. Taken together, the relationship between incarceration and health is complex.^74^ Therefore, one key area that public health research should focus on is whether and how incarceration impacts on health—positively or negatively—and better understand the mental and physical health trajectories of people before, during, and after imprisonment. The effects of mass incarceration need particular consideration in relation to health disparities and indirect consequences on families and communities.^75^ Longitudinal cohort studies with a population sampling frame, in which incarceration is treated as an exposure, might help clarify the multiple pathways linking incarceration and health.

Irrespective of the direction of causality and underlying mechanisms, our review clearly shows that people in prisons experience poor health across a wide range of mental and physical conditions. Therefore, national standards to meet the complex health needs of people in prisons should be implemented, evaluated, and periodically reviewed. Health improvements might be more effectively achieved through governance and service delivery models that integrate prison health care within the public health system, rather than being the responsibility of justice ministries.^76^ In accordance with the principle of equivalence of care, people in prisons should enjoy the same standards of health care that are available in the community, without discrimination on the grounds of their legal situation.^77^ However, targeting equivalent provision of services might be insufficient in this vulnerable population with complex and multifaceted health needs, and achieving equivalence of health outcomes might be a more appropriate objective.^78^ In practice, treatment coverage in prisons continues to be poor, and this treatment gap is probably more pronounced in LMICs than in HICs due to resource constraints.^79^ However, even in high-income settings, considerable differences exist in the provision of prison health care.^80^ Mental health services need to be adequately resourced and linked with evidence-based interventions^7^ to address the high level of unmet need in prison populations.^81^ In terms of treatment, cognitive behavioural approaches currently have the most consistent evidence in reducing depression and anxiety,^82^ although more high-quality trials are needed.^7^ Cost-effective and scalable interventions (eg, group-based formats) should be prioritised.^83^ The markedly higher prevalence of PTSD and drug misuse in women than in men indicates different mental health needs and underscores calls for a gender-sensitive approach to treatment.^84^ Trauma-informed care is widely discussed but has a relatively thin evidence-base in support.^85^ Although evidence on the effectiveness of psychosocial interventions for substance misuse in prison settings is mixed,^86^ robust data support opioid agonist treatment in reducing drug use and drug-related harms.^87^ There is a key role for primary care in prisons, which can lead to earlier treatment, better disease management, and quicker preventive care.^88^ For infectious diseases, active case finding through routine screening upon entry into prison (using opt-out testing strategies) could improve outcomes^89^ and allow for the scale-up of treatments, especially for the efficacious and well-tolerated direct-acting antiviral treatments for hepatitis C virus infection.^90^ Prisons have been identified as crucial sites for hepatitis C elimination campaigns.^91^ Evidence-based practices for HIV prevention (eg, pre-exposure prophylaxis) and treatment^92^ (eg, antiretroviral therapy) should be made available.^93^ Implementation of universal hepatitis B vaccination in prisons has been associated with an increased level of coverage among people who inject drugs in the community.^94^ Prisons could further offer the possibility to review and update vaccination schedules (eg, MMR and HPV) as part of a broader public health strategy.^95^ In general, strategies to facilitate linkage to and retention in post-release services, including discharge planning and transitional care coordination, are required to ensure maintenance of treatment gains.^96^

On a policy level, there is increasing recognition that drug use should be approached as a public health issue rather than a criminal justice issue.^97^ Decreasing incarceration rates might reduce infectious disease prevalence at the population level.^98^ Diversion from custody of people with severe mental illness to alternatives such as secure hospitals, community sentences, or treatment orders can be beneficial in terms of mental health and criminal justice outcomes.^99^ Structural interventions aimed at addressing the upstream causes of poor health (eg, poverty, unstable housing, and trauma) have further potential to improve outcomes in socially excluded populations.^100^ Policies intended to address health inequalities at the population level should be inclusive of the health needs of people in prisons. Overall, improving the health of people who experience incarceration will require structural funding and political will to provide appropriate health care in prisons.

The disproportionate burden of mental and physical disease in the prison population presents both challenges and opportunities. The prison context poses unique challenges to the delivery of health-care services, including security requirements.^101^ Frequent movements between prisons (often with no transfer of medical records) and short stays make engagement with health care difficult, and structural barriers such as overcrowding and understaffing further impede the optimal delivery of care.^102^ Additionally, individual-level barriers that prevent people in prisons from accessing available services include distrust of the health-care system, low health literacy and help-seeking behaviour, and fear of stigmatisation.^103^ Despite these challenges, incarceration provides a unique time window during which the multifaceted health needs of underserved populations can be assessed, diagnosed, and treated—often for the first time. Because almost all people in prisons will be released at some point, improving their health during imprisonment has the potential to equally improve the health of the communities to which they will return, hence producing a public health benefit.^104^ Treatment of mental illness and substance misuse might additionally contribute to public safety by decreasing rates of reoffending.^105^ In turn, these effects could lead to economic benefits by reducing the burden on health and criminal justice systems.^104^ In conclusion, incarceration provides an important opportunity to address unmet health needs in a vulnerable population, which can positively impact public health, public safety, and society as a whole.

## Data sharing

Data are available from the corresponding author on request.

## Declaration of interests

SF and JH are members of the UK's Independent Panel on Deaths in Custody. All other authors declare no competing interests.
