# Supplementary material for: Mental and physical health morbidity among people in prisons: an umbrella review
Source: Lancet Public Health. 2024 Mar 27;9(4):e250–60. doi: 10.1016/S2468-2667(24)00023-9 (PMC11652378; doi:10.1016/S2468-2667(24)00023-9)
Supplement: Supplementary appendix [file mmc1.pdf]

THE LANCET
Public Health

Supplementary appendix

This appendix formed part of the original submission and has been peer reviewed. We post it as supplied by the authors.

Supplement to: Favril L, Rich JD, Hard J, Fazel S. Mental and physical health morbidity among people in prisons: an umbrella review. *Lancet Public Health* 2024; **9:** e250–60.

Supplementary materials

Table S1	PRISMA checklist	p.2
Figure S1	Study selection	p.5
Table S2	List of excluded meta-analyses, with reason	p.6
Table S3	Overview of included meta-analyses	p.7
Table S4	List of LMICs included	p.8
Figure S2	Prevalence of drug use disorders by sex	p.9
Figure S3	Point (6-month) prevalence of PTSD by sex	p.10
Figure S4	Prevalence of antisocial personality disorder by sex	p.11
Figure S5	Prevalence of borderline personality disorder in women	p.12
Figure S6	Prevalence of epilepsy	p.13
Table S5	Prevalence of mental disorders by country income level	p.14
Box S1	Secondary analysis of mental health conditions	p.15
Table S6	Prevalence of comorbid mental illness and substance use disorder	p.16
Table S7	Prevalence of mental disorders by age group	p.17
Table S8	Prevalence of mental health conditions in LMICs by sex	p.18
Table S9	Prevalence of infectious diseases by sex	p.19
Box S2	Secondary analysis of physical health conditions	p.20
Box S3	Comparison with the general population	p.21
Box S4	Selection of overlapping meta-analyses: infectious diseases	p.22

Table S1. PRISMA checklist.

Section and Topic	Item #	Checklist item	Location where item is reported
**TITLE**			
Title	1	Identify the report as a systematic review.	NA
**ABSTRACT**			
Abstract	2	See the PRISMA 2020 for Abstracts checklist.	p.2
**INTRODUCTION**			
Rationale	3	Describe the rationale for the review in the context of existing knowledge.	p.4
Objectives	4	Provide an explicit statement of the objective(s) or question(s) the review addresses.	p.4
**METHODS**			
Eligibility criteria	5	Specify the inclusion and exclusion criteria for the review and how studies were grouped for the syntheses.	p.5
Information sources	6	Specify all databases, registers, websites, organisations, reference lists and other sources searched or consulted to identify studies. Specify the date when each source was last searched or consulted.	p.5
Search strategy	7	Present the full search strategies for all databases, registers and websites, including any filters and limits used.	p.5
Selection process	8	Specify the methods used to decide whether a study met the inclusion criteria of the review, including how many reviewers screened each record and each report retrieved, whether they worked independently, and if applicable, details of automation tools used in the process.	p.5
Data collection process	9	Specify the methods used to collect data from reports, including how many reviewers collected data from each report, whether they worked independently, any processes for obtaining or confirming data from study investigators, and if applicable, details of automation tools used in the process.	p.6
Data items	10a	List and define all outcomes for which data were sought. Specify whether all results that were compatible with each outcome domain in each study were sought (e.g. for all measures, time points, analyses), and if not, the methods used to decide which results to collect.	p.5
	10b	List and define all other variables for which data were sought (e.g. participant and intervention characteristics, funding sources). Describe any assumptions made about any missing or unclear information.	p.6
Study risk of bias assessment	11	Specify the methods used to assess risk of bias in the included studies, including details of the tool(s) used, how many reviewers assessed each study and whether they worked independently, and if applicable, details of automation tools used in the process.	p.7
Effect measures	12	Specify for each outcome the effect measure(s) (e.g. risk ratio, mean difference) used in the synthesis or presentation of results.	p.5

Section and Topic	Item #	Checklist item	Location where item is reported
Synthesis methods	13a	Describe the processes used to decide which studies were eligible for each synthesis (e.g. tabulating the study intervention characteristics and comparing against the planned groups for each synthesis (item #5)).	p.5
	13b	Describe any methods required to prepare the data for presentation or synthesis, such as handling of missing summary statistics, or data conversions.	p.5
	13c	Describe any methods used to tabulate or visually display results of individual studies and syntheses.	p.6
	13d	Describe any methods used to synthesize results and provide a rationale for the choice(s). If meta-analysis was performed, describe the model(s), method(s) to identify the presence and extent of statistical heterogeneity, and software package(s) used.	p.6
	13e	Describe any methods used to explore possible causes of heterogeneity among study results (e.g. subgroup analysis, meta-regression).	p.6
	13f	Describe any sensitivity analyses conducted to assess robustness of the synthesized results.	p.6
Reporting bias assessment	14	Describe any methods used to assess risk of bias due to missing results in a synthesis (arising from reporting biases).	p.6
Certainty assessment	15	Describe any methods used to assess certainty (or confidence) in the body of evidence for an outcome.	p.6
**RESULTS**			
Study selection	16a	Describe the results of the search and selection process, from the number of records identified in the search to the number of studies included in the review, ideally using a flow diagram.	p.7<br>Figure S1
	16b	Cite studies that might appear to meet the inclusion criteria, but which were excluded, and explain why they were excluded.	p.5
Study characteristics	17	Cite each included study and present its characteristics.	p.7<br>Table S3
Risk of bias in studies	18	Present assessments of risk of bias for each included study.	p.8
Results of individual studies	19	For all outcomes, present, for each study: (a) summary statistics for each group (where appropriate) and (b) an effect estimate and its precision (e.g. confidence/credible interval), ideally using structured tables or plots.	Table 1
Results of syntheses	20a	For each synthesis, briefly summarise the characteristics and risk of bias among contributing studies.	p.7-8
	20b	Present results of all statistical syntheses conducted. If meta-analysis was done, present for each the summary estimate and its precision (e.g. confidence/credible interval) and measures of statistical heterogeneity. If comparing groups, describe the direction of the effect.	p.8-9
	20c	Present results of all investigations of possible causes of heterogeneity among study results.	p.8-9
	20d	Present results of all sensitivity analyses conducted to assess the robustness of the synthesized results.	p.8-9

Section and Topic	Item #	Checklist item	Location where item is reported
Reporting biases	21	Present assessments of risk of bias due to missing results (arising from reporting biases) for each synthesis assessed.	p.8
Certainty of evidence	22	Present assessments of certainty (or confidence) in the body of evidence for each outcome assessed.	p.8
**DISCUSSION**			
Discussion	23a	Provide a general interpretation of the results in the context of other evidence.	p.9-11
	23b	Discuss any limitations of the evidence included in the review.	p.9-11
	23c	Discuss any limitations of the review processes used.	p.11
	23d	Discuss implications of the results for practice, policy, and future research.	p.11-13
**OTHER INFORMATION**			
Registration and protocol	24a	Provide registration information for the review, including register name and registration number, or state that the review was not registered.	p.4
	24b	Indicate where the review protocol can be accessed, or state that a protocol was not prepared.	p.4
	24c	Describe and explain any amendments to information provided at registration or in the protocol.	NA
Support	25	Describe sources of financial or non-financial support for the review, and the role of the funders or sponsors in the review.	p.2,14
Competing interests	26	Declare any competing interests of review authors.	p.14
Availability of data, code and other materials	27	Report which of the following are publicly available and where they can be found: template data collection forms; data extracted from included studies; data used for all analyses; analytic code; any other materials used in the review.	p.14

Figure S1. Study selection.

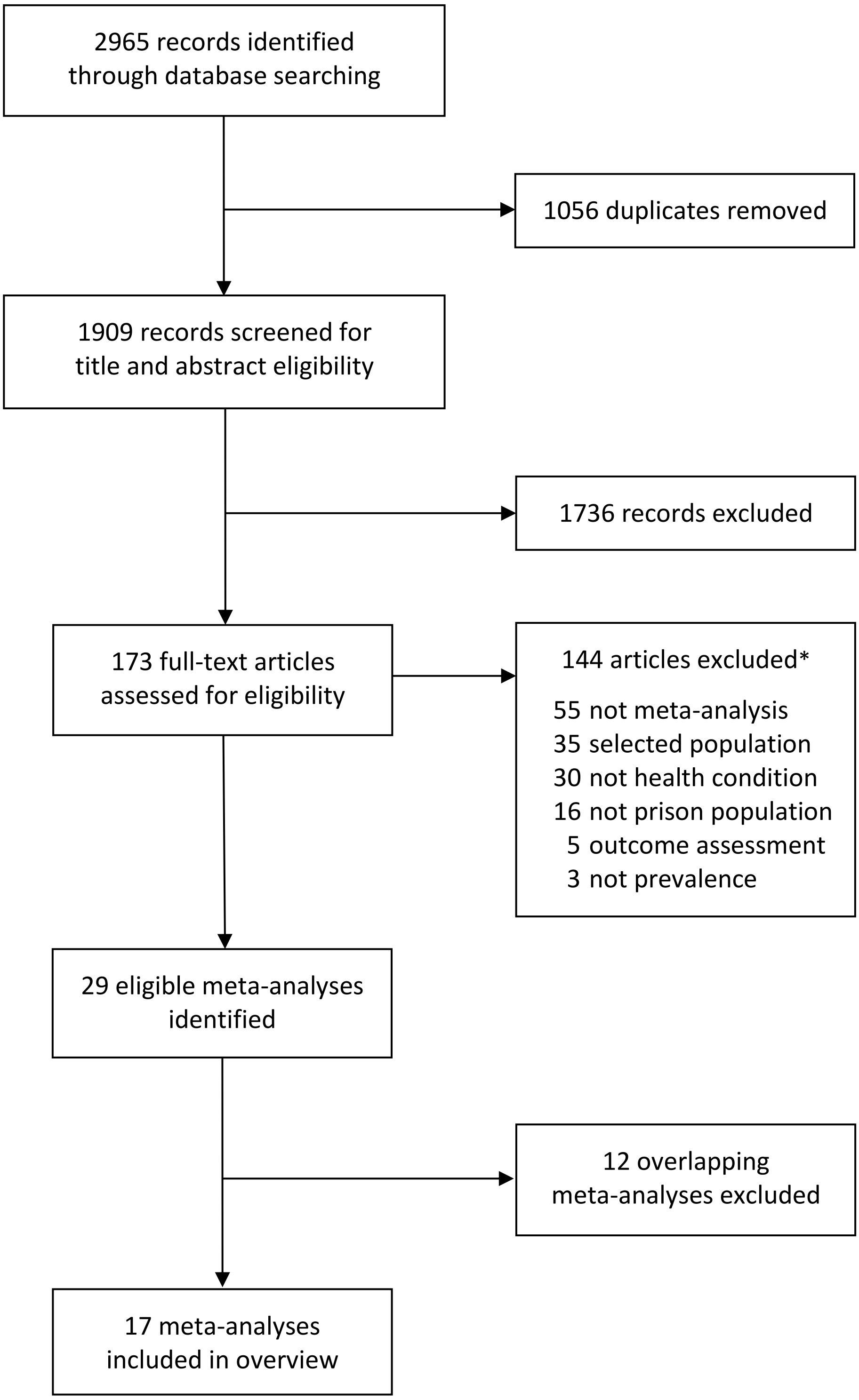

* Articles can be excluded for multiple reasons; numbers listed are based on the major reason for exclusion.

Table S2. List of excluded meta-analyses, with reason.

Meta-analysis	Condition	Reason for exclusion
Bedaso et al. (2020)	Depression	Higher quality review available (Fazel & Seewald, 2012)
Busschots et al. (2022)	Hepatitis C virus	Includes self-report, no pooled estimate reported
Cords et al. (2021)	Tuberculosis	Other closed settings, no study-level data available, no pooled estimate reported (prisons and jails combined)
Dolan et al. (2016)	Infectious diseases	Other closed settings, no study-level data available
Fazel et al. (2006)	Substance use disorder	Update available (Fazel et al., 2017)
Fazel et al. (2008)	Mental disorders	Update available (Beaudry et al., 2021)
Larney et al. (2013)	Hepatitis C virus	Other closed settings, no study-level data available
Livanou et al. (2019)	Mental disorders	Higher quality review available (Beaudry et al., 2021)
Moreira et al. (2019)	Tuberculosis	Higher quality review available (Placeres et al., 2023)
Moreira et al. (2022)	Tuberculosis	Higher quality review available (Placeres et al., 2023)
Vescio et al. (2008)	Hepatitis C virus	No pooled estimate reported, dated (studies until 2005)
Young et al. (2015)	ADHD	Update available (Fazel & Favril, 2023)

Table S3. Overview of included meta-analyses.

Meta-analysis	Health condition(s)	*k*	*n*	Date range	Countries (LMICs)	Age group	Mean age	% men	By sex	ROB
MAIN ANALYSIS										
Fazel & Seewald (2012)	Depression and psychotic illness	109	33,588	1966–2010	24 (8)	Adults	30.5	84	Yes	M
Fazel et al. (2017)	Substance use disorders	24	18,388	1988–2015	10 (0)	Adults	30.2	64	Yes	L
Baranyi et al. (2018)*	Posttraumatic stress disorder	50	19,011	1990–2017	20 (6)	Adults	30.6	76	Yes	L
Fazel & Favril (2023)	ADHD	11	3919	2010–2022	10 (3)	Adults	34.1	91	No	M
Fazel & Danesh (2002)†	Personality disorders	28	13,844	1987–1999	12 (0)	Adults	29	81	Yes	H
Salari et al. (2022)	Hepatitis C virus	93	145,823	1990–2020	35 (16)	Adults	33.8	NR	No	H
Moradi et al. (2018)	Hepatitis B virus	43	82,950	2005–2015	18 (11)	NR	NR	NR	Yes	H
Sayyah et al. (2019)	HIV	72	2,275,930	1989–2017	NR	Adults	NR	NR	No	H
Placeres et al. (2023)	Tuberculosis	74	NR	1986–2021	31 (24)	NR	NR	NR	No	L
Kouyoumdjian et al. (2012)	Sexually transmitted infections	60	NR	1985–2010	10 (6)	All	NR	NR	Yes	L
Escobar & Plugge (2020)	HPV and CIN	35	53,533	1968–2017	10 (4)	All	NR	0	—	M
Fazel et al. (2002)	Epilepsy	7	3111	1966–1998	4 (1)	Adults	29	90	No	H
SECONDARY ANALYSIS										
Munday et al. (2019)	Non-communicable diseases	26	93,862	1992–2016	11 (1)	>50y	NR	NR	No	L
Di Lorito et al. (2018)	Mental disorders	9	2447	1995–2016	3 (0)	>50y	NR	NR	No	H
Beaudry et al. (2021)	Mental disorders	47	32,787	1976–2019	19 (5)	10–19y	16	85	Yes	L
Baranyi et al. (2019)	Mental disorders	30	14,527	1997–2018	13 (13)	Adults	31.8	85	Yes	L
Baranyi et al. (2022)	Comorbid mental disorders	59	24,915	1988–2020	21 (9)	Adults	NR	69	No	L

Note. k, number of samples; *n*, pooled sample size; NR, not reported; LMICs, low-income and middle-income countries; ROB, risk of bias (L, low; M, moderate; H, high); ADHD, attention-deficit hyperactivity disorder; HIV, human immunodeficiency virus; HPV, human papilloma virus; CIN, cervical intraepithelial neoplasia.

* Numbers refer to 6-month prevalence only (the overall meta-analysis included a total of 56 studies with 21,099 individuals).

† Numbers refer to antisocial and borderline personality disorders only (the overall meta-analysis included a total of 62 studies with 22,790 individuals).

Table S4. List of LMICs included.

Health condition	LMICs
Psychotic illness	Brazil, India, Iran, Kuwait, Malaysia, Nigeria
Major depression	Brazil, Dubai (UAE), Iran, Kuwait, Malaysia, Mexico, Nigeria
PTSD	Brazil, China, India, Iran, South Africa, Turkey
Alcohol use disorder	*None included*
Drug use disorder	*None included*
ADHD	Brazil, Iran, South Africa
Antisocial personality disorder	*None included*
Borderline personality disorder	*None included*
Hepatitis C virus	Brazil, Egypt, Ethiopia, Ghana, Indonesia, Iran, Kyrgyzstan, Lebanon, Macedonia, Mexico, Nigeria, Pakistan, Senegal, Togo, Turkey, Ukraine
Hepatitis B virus	Azerbaijan, Brazil, Ghana, Indonesia, Iran, Lebanon, Macedonia, Mexico, Nigeria, Pakistan, Ukraine
HIV	*Not reported*
Tuberculosis	Bangladesh, Brazil, Cameroon, Colombia, Côte d'Ivoire, Congo, Ethiopia, India, Iran, Lebanon, Malawi, Malaysia, Mexico, Nepal, Nigeria, Pakistan, Peru, South Africa, Tajikistan, Thailand, Turkey, Uganda, Zambia, Zimbabwe
Chlamydia	Brazil, Jamaica, Pakistan
Gonorrhoea	Brazil, Jamaica, Pakistan
Syphilis	Brazil, Ghana, India, Jamaica, Mozambique
Human papillomavirus	Brazil, Mexico, Russia
Cervical intraepithelial neoplasia	Brazil, Mexico, Peru
Epilepsy	Nigeria
Overall	39 LMICs:<br>Azerbaijan (1), Bangladesh (1), Brazil (15), Cameroon (1), China (1), Colombia (1), Congo (1), Côte d'Ivoire (1), Dubai (1), Egypt (1), Ethiopia (2), Ghana (4), India (7), Indonesia (3), Iran (10), Jamaica (3), Kuwait (2), Kyrgyzstan (1), Lebanon (4), Macedonia (2), Malawi (1), Malaysia (5), Mexico (6), Mozambique (2), Nepal (1), Nigeria (8), Pakistan (5), Peru (3), Russia (1), Senegal (1), South Africa (3), Tajikistan (1), Thailand (1), Togo (1), Turkey (5), Uganda (1), Ukraine (4), Zambia (1), Zimbabwe (1)

Note that income classifications may have changed over time (eg, Dubai and Kuwait).

Figure S2. Prevalence of drug use disorders by sex (Fazel et al., 2017).

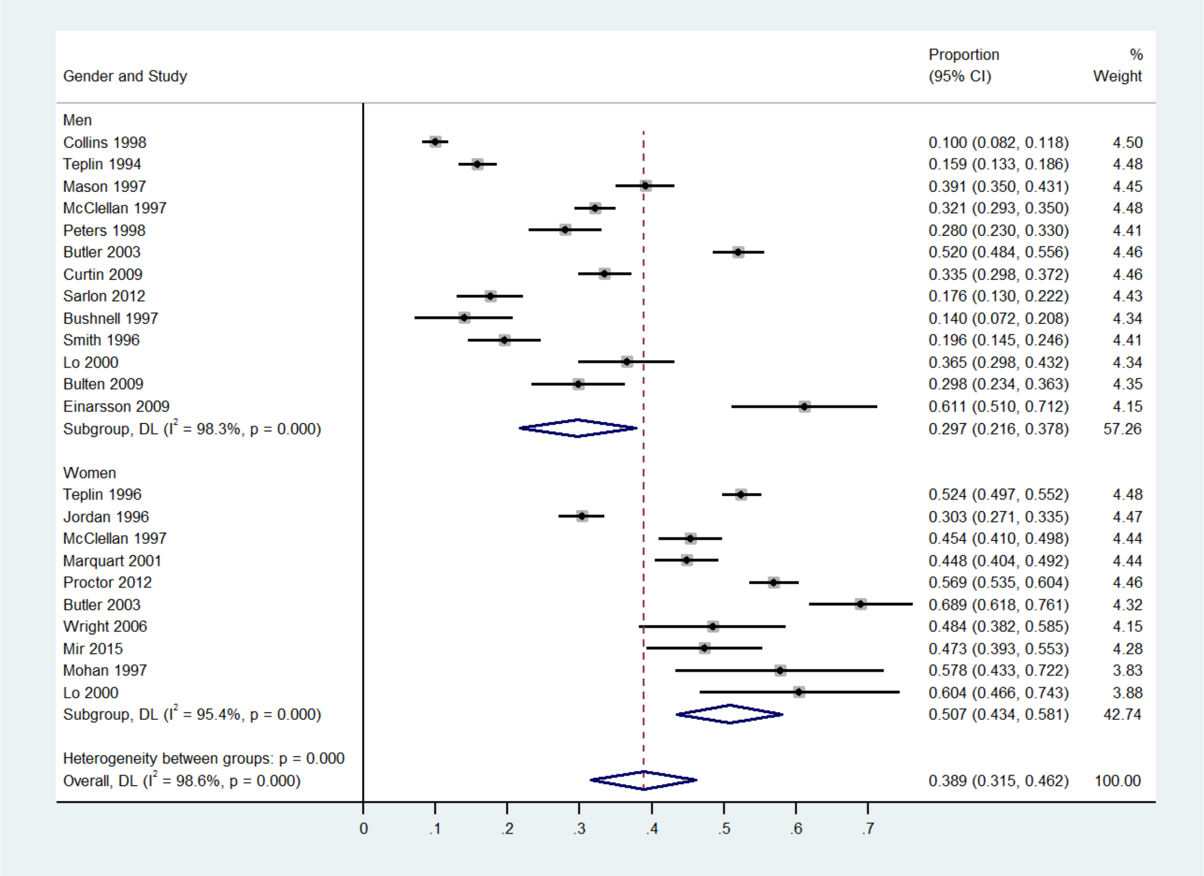

Figure S3. Point (6-month) prevalence of PTSD by sex (Baranyi et al., 2018).

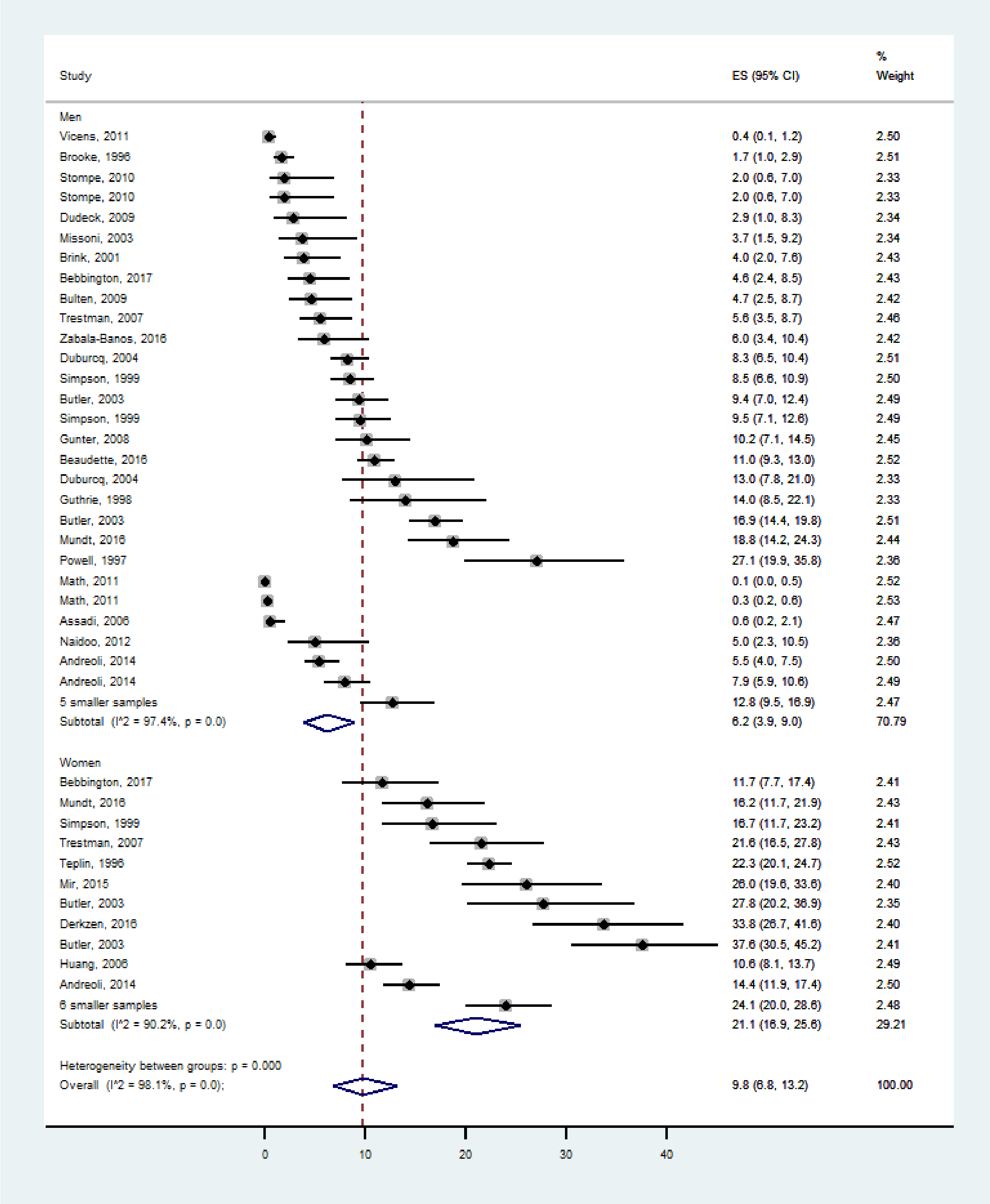

Figure S4. Prevalence of antisocial personality disorder by sex (Fazel & Danesh, 2002).

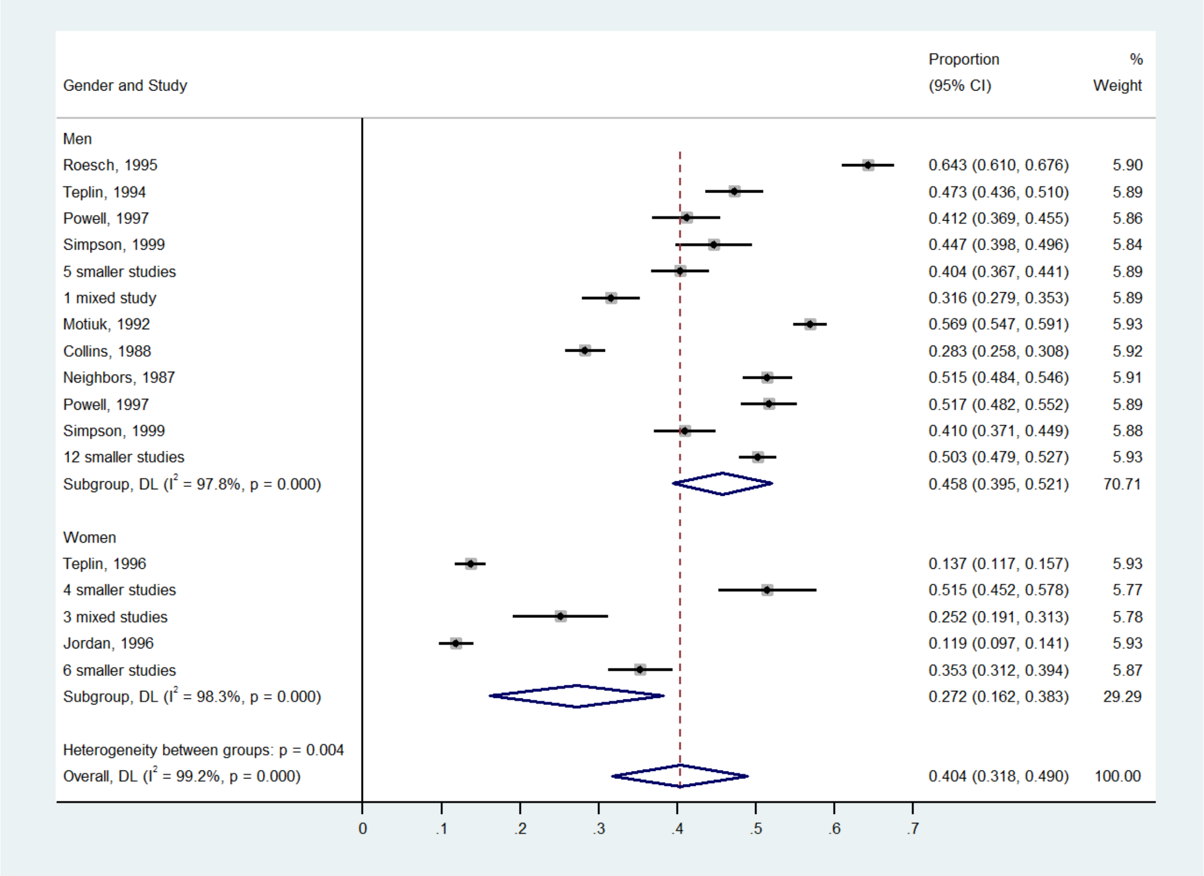

Note. In meta-regression, prevalence was significantly higher in men than women (p = 0.012).

Figure S5. Prevalence of borderline personality disorder in women (Fazel & Danesh, 2002).

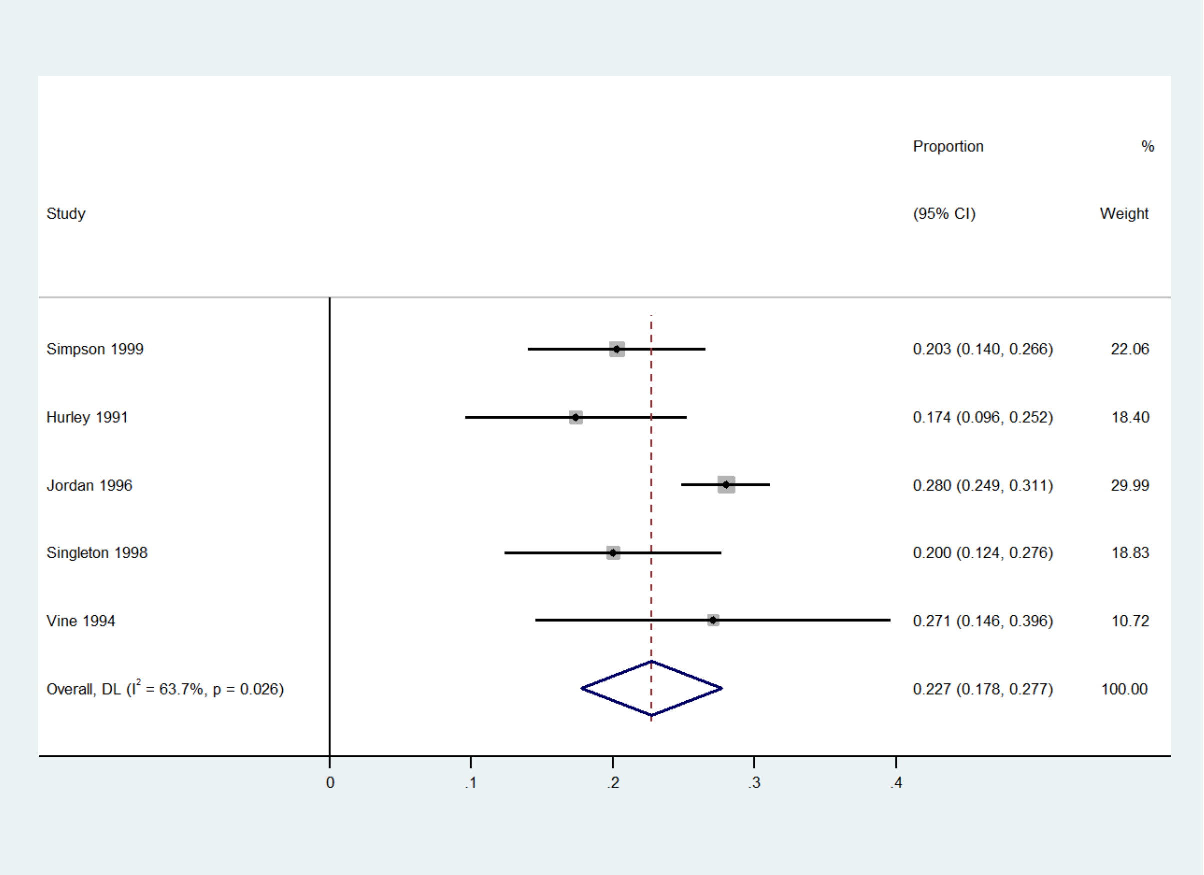

Figure S6. Prevalence of epilepsy (Fazel et al., 2002).

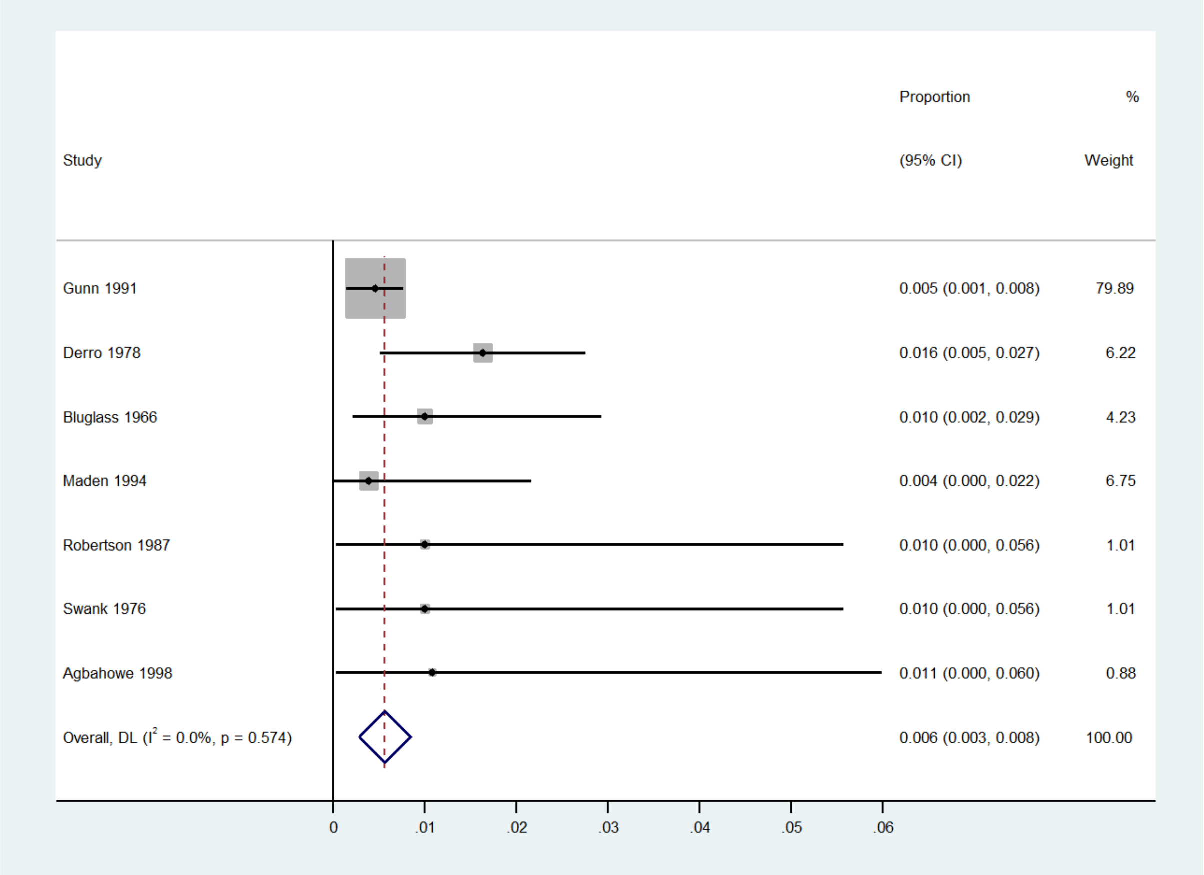

Table S5. Prevalence of mental disorders by country income level.

**Diagnosis**	**LMICs**	**HICs**
Psychotic illness	5.5 (4.2–6.8)	3.5 (3.0–3.9)
Major depression	22.5 (10.6–34.4)	10.0 (8.7–11.2)
Posttraumatic stress disorder	5.4 (1.9–10.3)	12.4 (9.4–15.7)

Note. As reported in the original meta-analyses (Baranyi et al., 2018; Fazel & Seewald, 2012), differences in prevalence between LMICs and HICs were significant in meta-regression analyses for all three mental disorders (p = 0.035 for psychotic illness, p = 0.001 for major depression, and p = 0.02 for posttraumatic stress disorder).

Box S1. Secondary analysis of mental health conditions.

Comorbidity

A meta-analysis of 50 studies (*n* = 24,915) examined current comorbidity between mental illness and substance use disorders (Baranyi et al., 2022). The pooled prevalence was 3.5% (2.2–5.0) for psychotic illness and comorbid substance use disorder, and 9.1% (5.6–13.3) for co-occurring major depression and substance use disorder (Table S6). Half of people with psychotic illness (49.2%) and major depression (51.6%) had a comorbid alcohol or drug use disorder. Across diagnoses, the lifetime prevalence of comorbidity was double that of current comorbidity. For example, on a lifetime basis, one in five (22.2%, 16.9–28.0) people in prisons had a comorbid major depression and substance use disorder. The current and lifetime prevalence of a co-occurring Axis I disorder and substance use disorder was 20.7% (13.8–28.5) and 39.9% (28.3–52.1), respectively.

Adolescents and older adults

Two meta-analyses examined the prevalence of mental disorders in specific age groups (Table S7). One review synthesised data from 47 studies comprising 32,787 adolescents (aged 10–19 years) in juvenile detention (Beaudry et al., 2021), documenting prevalences largely similar to those found in adults for psychotic illness (2.9%, 2.4–3.4), major depression (14.9%, 12.6–17.2), and PTSD (11.0%, 8.6–13.4), but markedly higher for ADHD (17.3%, 14.6–20.0). Lifetime prevalence of conduct disorder in adolescents was 61.0% (55.5–66.5). Girls had significantly higher prevalences of major depression and PTSD than boys; no sex differences were found for the other disorders under study (Beaudry et al., 2021). Another meta-analysis (with high risk of bias) on mental disorders in 2447 older people in prisons (aged 50 years and over) reported more variation in prevalences (Di Lorito et al., 2018), although these estimates were based on a small number of primary studies (range 3 to 9). Dementia was found to affect 3.3% of older people in prisons.

Low-income and middle-income countries

A meta-analysis (Baranyi et al., 2019) of 30 samples (*n* = 14,527) focused on mental disorders in 13 LMICs. For men and women combined, the 12-month prevalence of psychotic illness and major depression was 6.2% (4.0–8.6) and 16.0% (11.7–20.8), respectively. Excluding admission studies, the 12-month prevalence was 3.8% (1.2–7.6) for alcohol use disorder and 5.1% (2.9–7.8) for drug use disorder in cross-section prison samples. Prevalence of mental illness and substance misuse in LMICs was broadly similar for incarcerated men and women (Table S8).

Table S6. Prevalence of comorbid mental illness and substance use disorder.

**Current comorbidity**	Major depression	Psychotic illness	Axis I disorder*
Substance use disorder	9.1 (5.6–13.3)	3.5 (2.2–5.0)	20.7 (13.8–28.5)
Alcohol use disorder	5.1 (3.1–7.7)	1.8 (1.1–2.7)	11.1 (6.6–16.5)
Drug use disorder	5.4 (2.7–8.9)	2.4 (1.2–4.0)	15.8 (8.9–24.2)
**Lifetime comorbidity**	Major depression	Psychotic illness	Axis I disorder*
Substance use disorder	22.2 (16.9–28.0)	6.9 (4.7–9.4)	39.9 (28.3–52.1)
Alcohol use disorder	12.4 (7.9–17.8)	4.9 (2.4–8.0)	27.9 (16.0–41.7)
Drug use disorder	14.3 (9.5–19.8)	5.3 (2.3–9.4)	29.7 (14.3–47.9)

Note. Prevalence estimates and 95% CI as reported in the original meta-analysis (Baranyi et al., 2022).
* Axis I disorders include International Classification of Diseases (ICD) codes F20–59.

Table S7. Prevalence of mental disorders by age group.

**Diagnosis**	**Adolescents (10–19 years)**	**Older adults (≥50 years)**
Psychotic illness	2.9 (2.4–3.4)	5.5 (5.3–5.7)
Major depression	14.9 (12.6–17.2)	28.3 (27.8–28.8)
PTSD	11.0 (8.6–13.4)	6.2 (6.0–6.4)
Conduct disorder	61.0 (55.5–66.5)	—
ADHD	17.3 (14.6–20.0)	—
Bipolar disorder	—	4.5 (4.4–4.6)
Alcohol use disorder	—	15.9 (14.6–17.2)
Anxiety disorder	—	14.2 (13.6–14.7)
Personality disorder	—	22.9 (22.4–23.4)
Dementia	—	3.3 (3.2–3.4)

Note. Prevalence estimates and 95% CI as reported in the original meta-analyses of adolescents (boys and girls combined; Beaudry et al., 2021) and older adults (Di Lorito et al., 2018).

Table S8. Prevalence of mental health conditions in LMICs by sex.

**Diagnosis**	**Men**	**Women**
Psychotic illness	6.6 (3.7–10.2)	5.7 (1.9–11.0)
Major depression	15.9 (11.1–21.4)	19.4 (11.7–28.5)
Alcohol use disorder*	3.7 (0.5–9.4)	4.4 (1.5–8.4)
Drug use disorder*	5.3 (2.5–9.0)	5.0 (1.6–9.8)

Note. Prevalence estimates and 95% CI as reported in the original meta-analysis (Baranyi et al., 2019).
* Non-admission (cross-sectional) samples only.

Table S9. Prevalence of infectious diseases by sex.

Infectious disease	Men	Women
Hepatitis B virus	6.7 (6.5–6.9)	4.3 (4.0–4.8)
Chlamydia	5.8 (5.0–6.5)	12.3 (10.6–14.0)
Gonorrhoea	1.4 (1.1–1.7)	5.7 (4.8–6.7)
Syphilis	2.5 (2.1–2.8)	6.1 (4.8–7.5)

Note. Prevalence estimates and 95% CI as reported in the original meta-analyses (Kouyoumdjian et al., 2012; Moradi et al., 2018).

Box S2. Secondary analysis of physical health conditions.

A meta-analysis (Munday et al., 2019) on non-communicable diseases in 93,862 older people in prisons (aged 50 years and over) reported high prevalences of cancer (8%, 6–10), cardiovascular disease (38%, 33–42), hypertension (39%, 32–47), ischaemic heart disease (20%, 16–24), diabetes (14%, 12–16), asthma (7%, 5–10), chronic obstructive pulmonary disease (8%, 5–11), and arthritis (37%, 24–51). When compared to younger people (aged less than 50 years) in prisons, disease prevalence was consistently higher in older prison populations (eg, chronic obstructive pulmonary disease was reported to be eight times higher in older compared to younger people in prisons, diabetes 10 times, ischaemic heart disease 13 times, and hypertension over 20 times).

Box S3. Comparison with the general population.

When comparing cervical intraepithelial neoplasia prevalence in imprisoned women to the general population, all studies included in the meta-analysis (Escobar & Plugge, 2020) showed prevalence ratios greater than one (ranging between 1.1 and 5.5), indicating a higher prevalence in imprisoned women than in the community. For mental disorders, prevalence ratios indicated substantially higher rates of psychotic illness (15.8), major depression (6.0), drug use disorders (6.1), and alcohol use disorder (2.4) among people incarcerated in LMICs than in the general population (Baranyi et al., 2019). Compared with older people in the community, relative risks indicated that older people in prisons were more likely to have any mental disorder (2.5), depression (2.8), and personality disorder (2.3), while increased yet non-significant relative risks (ranging between 1.3 and 6.0) were found for schizophrenia, bipolar disorder, PTSD, alcohol use disorder, and anxiety disorders (Di Lorito et al., 2018). For non-communicable diseases, pooled prevalence rates were at least double among older people in prisons compared to similar age groups in the community (Munday et al., 2019). For other health conditions, the relative excess in prevalence was not examined directly, although these were thought to be mostly higher. For example, the 6-month (point) prevalence of PTSD was found to be five times higher in incarcerated men and eight times higher in women compared with the general population of similar sex (Baranyi et al., 2018). In HICs, adults in prisons were ten times more likely to have antisocial personality disorder than their peers in the general population (Fazel & Danesh, 2002). The prevalence of ADHD was three times higher in prisons than in the general adult population (Fazel & Favril, 2023). Detained adolescents had consistently higher prevalences (at least double) of psychotic illness, major depression, ADHD, PTSD, and conduct disorder compared with age-equivalent community persons (Beaudry et al., 2021). One exception was epilepsy, for which the prevalence was reported to be similar between general and prison populations (Fazel et al., 2002).

Box S4. Selection of overlapping meta-analyses: infectious diseases.

When multiple eligible meta-analyses evaluated the same health condition, we retained the one with the highest quality, provided that individual-level study estimates were available to allow for quantitative analysis. In doing so, we were not able to include findings from high-quality meta-analyses on infectious diseases (Cords et al., 2021; Dolan et al., 2016) because the authors did not respond to requests for additional data or were unable to provide these. As a result, the included meta-analyses on infectious diseases (Moradi et al., 2018; Salari et al., 2022; Sayyah et al., 2019) were of low quality (high risk of bias based on ROBIS), although prevalence estimates were largely similar across included and excluded meta-analyses. For example, in the meta-analysis conducted by Dolan et al. (2016), the pooled prevalence was 15.1% (13.7–16.5) for hepatitis C virus, 4.8% (3.2–6.8) for hepatitis B virus, 3.8% (3.2–4.5) for HIV, and 2.8% (2.1–3.7) for active tuberculosis.

Baranyi, G., Cassidy, M., Fazel, S., Priebe, S., & Mundt, A.P. (2018). Prevalence of posttraumatic stress disorder in prisoners. *Epidemiologic Reviews*, *40*(1), 134-145.

Baranyi, G., Fazel, S., Langerfeldt, S.D., & Mundt, A.P. (2022). The prevalence of comorbid serious mental illnesses and substance use disorders in prison populations: a systematic review and meta-analysis. *Lancet Public Health*, *7*(6), e557-e568.

Baranyi, G., Scholl, C., Fazel, S., Patel, V., Priebe, S., & Mundt, A.P. (2019). Severe mental illness and substance use disorders in prisoners in low-income and middle-income countries: a systematic review and meta-analysis of prevalence studies. *Lancet Global Health*, *7*(4), e461-e471.

Beaudry, G., Yu, R., Langstrom, N., & Fazel, S. (2021). An updated systematic review and meta-regression analysis: mental disorders among adolescents in juvenile detention and correctional facilities. *Journal of the American Academy of Child and Adolescent Psychiatry*, *60*(1), 46-60.

Bedaso, A., Ayalew, M., Mekonnen, N., & Duko, B. (2020). Global estimates of the prevalence of depression among prisoners: a systematic review and meta-analysis. *Depression Research and Treatment*, *2020*, 3695209.

Busschots, D., Kremer, C., Bielen, R., Koc, O.M., Heyens, L., Nevens, F., et al. (2022). Hepatitis C prevalence in incarcerated settings between 2013–2021: a systematic review and meta-analysis. *BMC Public Health*, *22*(1), 2159.

Cords, O., Martinez, L., Warren, J.L., O'Marr, J.M., Walter, K.S., Cohen, T., et al. (2021). Incidence and prevalence of tuberculosis in incarcerated populations: a systematic review and meta-analysis. *Lancet Public Health*, *6*(5), e300-e308.

Di Lorito, C., Vollm, B., & Dening, T. (2018). Psychiatric disorders among older prisoners: a systematic review and comparison study against older people in the community. *Aging & Mental Health*, *22*(1), 1-10.

Dolan, K., Wirtz, A.L., Moazen, B., Ndeffo-mbah, M., Galvani, A., Kinner, S.A., et al. (2016). Global burden of HIV, viral hepatitis, and tuberculosis in prisoners and detainees. *Lancet*, *388*(10049), 1089-1102.

Escobar, N., & Plugge, E. (2020). Prevalence of human papillomavirus infection, cervical intraepithelial neoplasia and cervical cancer in imprisoned women worldwide: a systematic review and meta-analysis. *Journal of Epidemiology and Community Health*, *74*(1), 95-102.

Fazel, S., Bains, P., & Doll, H. (2006). Substance abuse and dependence in prisoners: a systematic review. *Addiction*, *101*(2), 181-191.

Fazel, S., & Danesh, J. (2002). Serious mental disorder in 23 000 prisoners: a systematic review of 62 surveys. *Lancet*, *359*(9306), 545-550.

Fazel, S., Doll, H., & Langstrom, N. (2008). Mental disorders among adolescents in juvenile detention and correctional facilities: a systematic review and metaregression analysis of 25 surveys. *Journal of the American Academy of Child and Adolescent Psychiatry*, *47*(9), 1010-1019.

Fazel, S., & Favril, L. (2023). Prevalence of ADHD in adult prisoners: an updated meta-analysis. *PsyArXiv*.

Fazel, S., & Seewald, K. (2012). Severe mental illness in 33 588 prisoners worldwide: systematic review and meta-regression analysis. *British Journal of Psychiatry*, *200*(5), 364-373.

Fazel, S., Vassos, E., & Danesh, V. (2002). Prevalence of epilepsy in prisoners: systematic review. *BMJ*, *324*(7352), 1495.

Fazel, S., Yoon, I.A., & Hayes, A.J. (2017). Substance use disorders in prisoners: an updated systematic review and meta-regression analysis in recently incarcerated men and women. *Addiction*, *112*(10), 1725-1739.

Kouyoumdjian, F.G., Leto, D., John, S., Henein, H., & Bondy, S. (2012). A systematic review and meta-analysis of the prevalence of chlamydia, gonorrhoea and syphilis in incarcerated persons. *International Journal of STD & AIDS*, *23*(4), 248-254.

Larney, S., Kopinski, H., Beckwith, C.G., Zaller, N.D., Des Jarlais, D., Hagan, H., et al. (2013). Incidence and prevalence of hepatitis C in prisons and other closed settings: results of a systematic review and meta-analysis. *Hepatology*, *58*(4), 1215-1224.

Livanou, M., Furtado, V., Winsper, C., Silvester, A., & Singh, S.P. (2019). Prevalence of mental disorders and symptoms among incarcerated youth: a meta-analysis of 30 studies. *International Journal of Forensic Mental Health*, *18*(4), 400-414.

Moradi, G., Goodarzi, E., & Khazaei, Z. (2018). Prevalence of Hepatitis B and C in prisons worldwide: a meta-analysis during the years 2005-2015. *Biomedical Research and Therapy*, *5*(4), 2235-2251.

Moreira, T.R., Lemos, A.C., Colodette, R.M., Gomes, A.P., & Batista, R.S. (2019). Prevalence of tuberculosis in incarcerated populations: systematic review and meta-analysis. *Pan American Journal of Public Health*, *43*, e16.

Moreira, T.R., Passos, I.B.J., Bueno, J.V.L., Maffacciolli, R., Colodette, R.M., & Miguel, P.S. (2022). Prevalence of multidrug-resistant tuberculosis in prisons: systematic review and meta-analysis. *Indian Journal of Medical Microbiology*, *40*(2), 193-199.

Munday, D., Leaman, J., O'Moore, E., & Plugge, E. (2019). The prevalence of non-communicable disease in older people in prison: a systematic review and meta-analysis. *Age and Ageing*, *48*(2), 204-212.

Placeres, A.F., Soares, D.D., Delpino, F.M., Moura, H.S.D., Scholze, A.R., dos Santos, M.S., et al. (2023). Epidemiology of TB in prisoners: a metanalysis of the prevalence of active and latent TB. *BMC Infectious Diseases*, *23*(1), 20.

Salari, N., Darvishi, N., Hemmati, M., Shohaimi, S., Ghyasi, Y., Hossaini, F., et al. (2022). Global prevalence of hepatitis C in prisoners: a comprehensive systematic review and meta-analysis. *Archives of Virology*, *167*(4), 1025-1039.

Sayyah, M., Rahim, F., Kayedani, G.A., Shirbandi, K., & Saki-Malehi, A. (2019). Global view of HIV prevalence in prisons: a systematic review and meta-analysis. *Iranian Journal of Public Health*, *48*(2), 217-226.

Vescio, M.F., Longo, B., Babudieri, S., Starnini, G., Carbonara, S., Rezza, G., et al. (2008). Correlates of hepatitis C virus seropositivity in prison inmates: a meta-analysis. *Journal of Epidemiology and Community Health*, *62*(4), 305-313.

Young, S., Moss, D., Sedgwick, O., Fridman, M., & Hodgkins, P. (2015). A meta-analysis of the prevalence of attention deficit hyperactivity disorder in incarcerated populations. *Psychological Medicine*, *45*(2), 247-258.
